# Supplementary material for: Construction and Validation of a Reliable Disulfidptosis-Related LncRNAs Signature of the Subtype, Prognostic, and Immune Landscape in Colon Cancer
Source: Int J Mol Sci. 2023 Aug 18;24(16):12915. doi: 10.3390/ijms241612915 (PMC10454603; doi:10.3390/ijms241612915)
Supplement: Supplementary file 1 [file ijms-24-12915-s001.zip › ijms-2540164-supplementary.pdf]

Supplementary Material

Construction and Validation of a Reliable Disulfidptosis-Related LncRNAs Signature of the Subtype, Prognostic, and Immune Landscape in Colon Cancer

Supplementary Table S1: Primers used for RT-qPCR.

Genes	Sequence（5'−3')
AP003555.1	CACAACCATGAAAACGCCCA
	CACTGTCCCTTTTGGCTCGT
ATP2B1.AS1	GCTCTGACGTCTGTGTTTCCA
	AAGTGAAGGGCGTCCCACT
AC007728.3	CGTATCAGGAACAACACCAAATCAG
	TCCAAAGGGCAAAACCTCACAT
NSMCE1.DT	CCTATTAGTAGACCAAGAAGCATCAC
	TGGGATTAGTAGCTGAAAGTATTCG
GAPDH	GGAGCGAGATCCCTCCAAAAT
	GGCTGTTGTCATACTTCTCATGG

Supplementary Table S2: the results of univariate Cox regression analysis.

genes	Hazard.Ratio	HR.95L	HR.95H	CI95	P.value
AC245041.1	1.6	1.2654932	2.031494	1.27−2.03	0.001
LINC01679	4.81	1.9357315	11.94121	1.94−11.94	0.001
AP003555.1	1.43	1.1475402	1.778153	1.15−1.78	0.001
NSMCE1.DT	19.23	2.8509712	129.6826	2.85−129.68	0.002
ATP2B1.AS1	7.75	2.1506324	27.91335	2.15−27.91	0.002

SNHG16	0.8	0.6886264	0.925684	0.69−0.93	0.003
AC009549.1	3.48	1.5260413	7.930183	1.53−7.93	0.003
AC093849.2	1.76	1.1710397	2.631223	1.17−2.63	0.006
MACORIS	4.25	1.4980318	12.05631	1.5−12.06	0.007
AL391422.4	1.87	1.1774839	2.983203	1.18−2.98	0.008
AC007541.1	5.35	1.5173615	18.87713	1.52−18.88	0.009
AC138207.5	1.36	1.0672454	1.732098	1.07−1.73	0.013
LINC02381	1.78	1.1075305	2.862665	1.11−2.86	0.017
AL513550.1	1.4	1.0538624	1.853076	1.05−1.85	0.02
GIHCG	4.28	1.2486481	14.69193	1.25−14.69	0.021
AC008972.2	2.01	1.1088717	3.633054	1.11−3.63	0.021
FAM160A1.DT	0.62	0.4078211	0.948008	0.41−0.95	0.027
AC009951.6	6.1	1.2296898	30.21395	1.23−30.21	0.027
LINC00861	1.82	1.058902	3.125971	1.06−3.13	0.03
PCAT1	0.2	0.0472184	0.868186	0.05−0.87	0.032
AC121757.1	2.01	1.059224	3.828298	1.06−3.83	0.033
AL355312.2	0.04	0.0016067	0.790039	0−0.79	0.035
PCED1B.AS1	1.35	1.019366	1.797102	1.02−1.8	0.036
AC000061.1	0.53	0.2887402	0.966476	0.29−0.97	0.038

AC015819.1	2.38	1.0510756	5.385198	1.05−5.39	0.038
AC006213.7	4.12	1.0836639	15.67085	1.08−15.67	0.038
AL109741.1	2.33	1.0433653	5.224199	1.04−5.22	0.039
AL390195.1	0.29	0.0887579	0.948047	0.09−0.95	0.041
AL512656.1	0.19	0.0369513	0.939556	0.04−0.94	0.042
AP002336.2	0.44	0.2015363	0.97108	0.2−0.97	0.042
AC007728.3	0.12	0.0151175	0.935634	0.02−0.94	0.043
U91328.1	2.28	1.0281582	5.048758	1.03−5.05	0.043
AC069243.1	0	4.36E−09	0.843671	0−0.84	0.046
CCAT2	0.03	0.0010705	0.986416	0−0.99	0.049

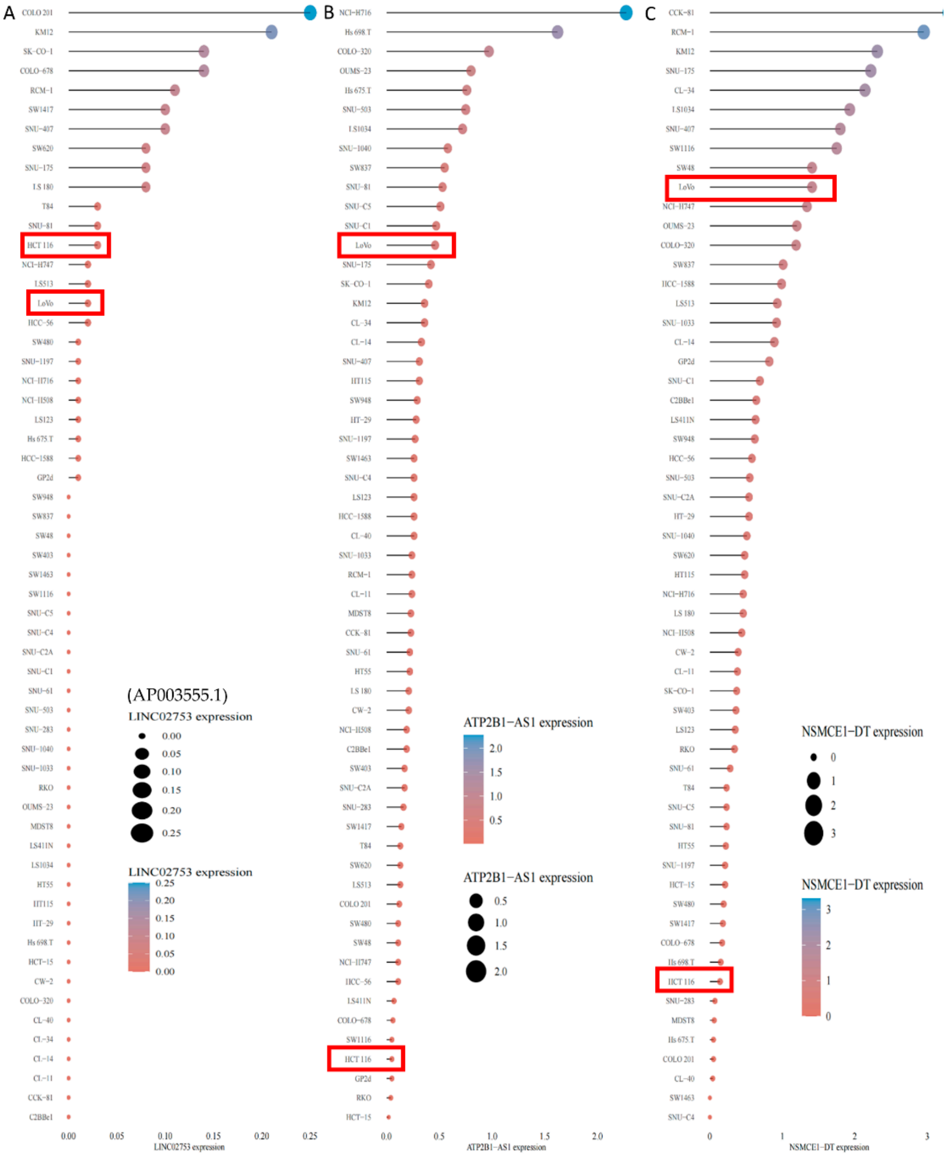

Figure S1. The cell line LncRNA expression matrix of COAD obtained from the CCLE dataset. (A) The expression distribution of AP003555.1 in different cell lines. (B) The expression distribution of ATP2B1.AS1 in different cell lines. (C) The expression distribution of NSMCE1.DT in different cell lines.
